# Predicting the current and future global distribution of the invasive freshwater hydrozoan *Craspedacusta sowerbii*

**DOI:** 10.1038/s41598-021-02525-3

**Published:** 2021-11-29

**Authors:** Guillaume Marchessaux, Florian Lüskow, Gianluca Sarà, Evgeny A. Pakhomov

**Affiliations:** 1 Department of Earth and Marine Science, University of Palermo, Viale delle Scienze, 90128 Palermo, Italy; 2 Department of Earth, Ocean and Atmospheric Sciences, University of British Columbia, 2039−2207 Main Mall, Vancouver, BC V6T 1Z4 Canada; 3 Institute for the Oceans and Fisheries, University of British Columbia, 2202 Main Mall, Vancouver, BC V6T 1Z4 Canada; 4 Hakai Institute, PO Box 309, Heriot Bay, BC V0P 1H0 Canada

**Keywords:** Invasive species, Biogeography, Climate change

## Abstract

The freshwater jellyfish *Craspedacusta sowerbii* is one of the most widespread invasive species, but its global distribution remains uncertain due to ephemeral appearances and general lack of information in various aquatic environments. The aim of this study was to map current and future distributions (2050 and 2100) using Species Distribution Models allowing to visualize the habitat suitability and make projections of its changes under potential climate change scenarios. Except in Oceania where the range decreased, an expansion of *C. sowerbii* was projected during the next century under modeled future scenarios being most intensive during the first half of the century. The present study shows that the expansion of *C. sowerbii* worldwide would be facilitated mainly by precipitation, vapor pressure, and temperature. The predictions showed that this species over the eighty years will invade high-latitude regions in both hemispheres with ecological consequences in already threatened freshwater ecosystems.

## Introduction

Non-indigenous species (NIS) constitute a major source of biological pollution, as some become invasive and have significant ecological and economic impacts on the biodiversity^1^, the ecosystem functioning (e.g., competition, predation, carbon flux), and human activities (e.g., fisheries, industrial complex, and tourism). The freshwater jellyfish *Craspedacusta sowerbii* (Hydrozoa, Limnomedusae, Olindiidae) is native to East Asia^2^ and was mainly introduced with aquatic plants such as the water lily *Victoria regia* into Europe in the 1850s, which was imported to botanic gardens in London and France^3^. Since the end of the nineteenth century, *C. sowerbii* was observed across the globe (e.g., Europe, North and South America, Australia, Asia, and parts of Africa)^4^.

Environmental factors such as temperature and food are main drivers in addressing sexual (by medusae) and asexual (by polyps) reproduction^5–9^. It has been documented that frustules develop into polyps between 12 and 20 °C^10,11^ and that the budding of medusae generally occurs at temperatures between 26 and 33 °C^5–7,11^. Under unfavorable environmental conditions (e.g., low temperatures and food concentration), formation of resistant polyps (e.g., podocysts) has been observed, which can survive up to forty years of desiccation^12^.

Despite its occasional and short-lived presence in the water column of invaded water bodies and potentially limited ecosystem impact, *C. sowerbii* has been intensively studied over 140 years after its discovery. Even if the biology is well known, the global distribution of *C. sowerbii* remains uncertain due to its ephemeral appearances and general lack of information in various aquatic environments. A single map of the worldwide distribution of *C. sowerbii* was published in 1994^13^, whereas a more recent summary of the genus *Craspedacusta* was published in 2008^2^. Globally, *C. sowerbii* inhabits very diverse habitats, ranging from small ponds to lakes, and there is an ongoing debate about species preferences, artificial vs natural water bodies^13^. The secondary dispersal of *C. sowerbii*, like many other NIS, was likely primarily facilitated through bird migrations, import of decorative aquatic plants and pet animals, as well as via small crafts carrying the polyps or encrusting stages^14^. *Craspedacusta sowerbii* can thrive in aquatic ecosystems with diverse environmental conditions^4,15^ and its proliferation is often explosive and short in duration^16,17^. It has been hypothesized that the occurrence of *C. sowerbii* will increase as a result of climate change introducing milder winters as well as warmer and longer summers^18^.

At present, there is neither a study describing the current global distribution of *C. sowerbii* nor predicting its range expansion under climate change scenarios. Such a study is needed to better understand potential effects of *C. sowerbii* on various freshwater systems. The aim of this study was to map current and future distributions of *C. sowerbii* using Species Distribution Models (SDMs) allowing to visualize the species geographic distribution based on the habitat suitability and to make projections of its changes under potential climate change scenarios.

## Results

**Figure 1:**
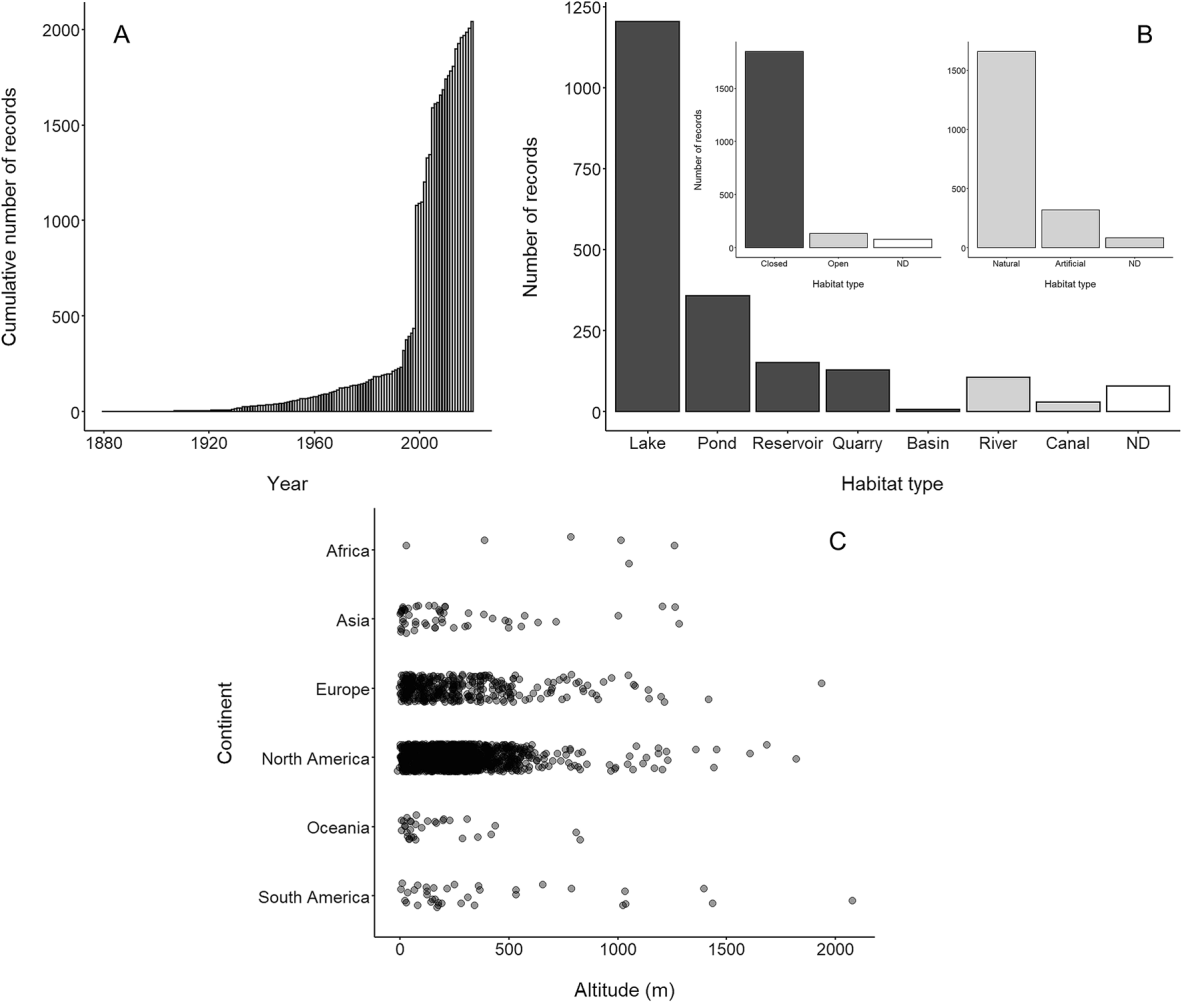
(**A**) Succession of the cumulative number of *Craspedacusta sowerbii* records worldwide, (**B**) habitat affinities (dark grey: closed systems, light grey: open systems, white: no data; ND), and (**C**) altitude of freshwater systems with *C. sowerbii*.

The number of *Craspedacusta sowerbii* records increased from 1 record in 1880 to 196 in 1988, and drastically increased to 2041 records in 2020 (Fig. 1A). Information on habitat types recorded in the literature showed that *C. sowerbii* was commonly found in natural (1659 records) environments, especially in closed systems (1847 records; i.e., lakes: 1204 records; ponds: 357 records; reservoirs: 151 records, and water-filled quarries: 129 records; Fig. 1B). *Craspedacusta sowerbii* was observed in freshwater systems in altitudes ranging from − 12 to 2076 m above sea level (Fig. 1C).

**Figure 2:**
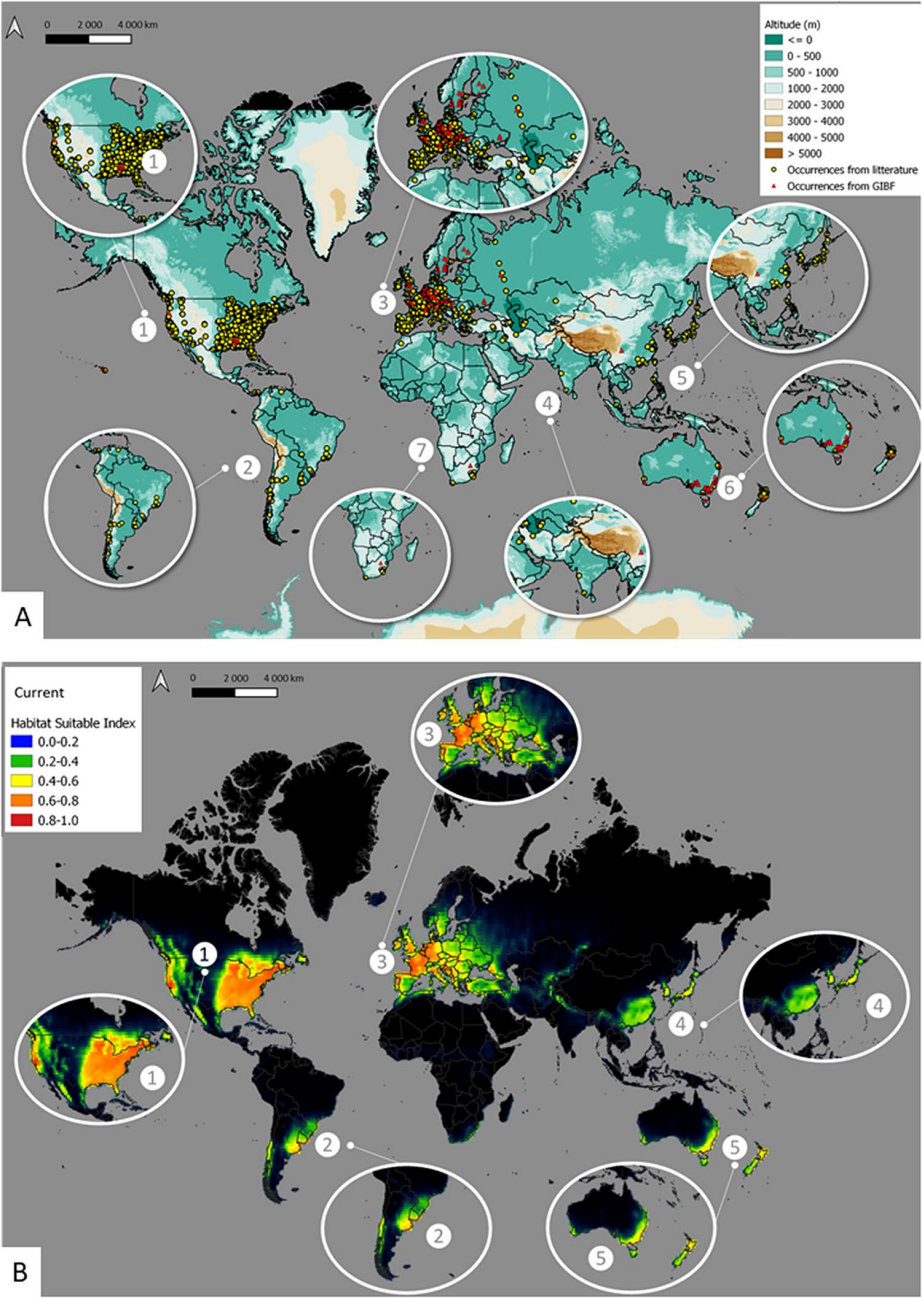
Distribution of *Craspedacusta sowerbii* (**A**) current occurrences and (**B**) predicted distribution under current climate conditions using Maxent at the global scale. Map produced using QGIS version 3.16.3 software, https://www.qgis.org/fr/site/index.html. Occurrences (graph A) were extracted from the database PANGAEA^64^.

*Craspedacusta sowerbii* occurs over a large range of latitudes and longitudes at the global scale (Fig. 2A) and this species is present on all continents, except Antarctica. North America and Europe were the most heavily invaded continents with 1525 records and 477 records, respectively (in comparison: 7 records in Africa, 40 records in South America, 53 records in Asia, 59 records in Oceania).

Predictions were consistent as all models had exceptional Area Under the Curve (AUC) values (> 0.9) and excellent True Skilled Statistics (TSS) (> 0.7) with low error values (Table 1A). Analysis of the unimodal environmental response curves indicated that the environmental conditions were within appropriate ranges for each epoch/scenario (see Supplementary Figs. S2 to S9). All environmental variables were found to be within their training ranges.

**Table 1:** (**A**) Model parameters and evaluation indicators. Area under the curve (AUC) and True Skilled Statistics (TSS), and standard deviation (SD) obtained for each scenario, and (**B**) percentage of contribution of environmental variables on suitable habitats for *C. sowerbii* under current scenario.

A		
Models	AUC ± SD	TSS ± SD
Current|	0.935 ± 0.003	0.81 ± 0.02
Future|2050–SSP126	0.914 ± 0.004	0.70 ± 0.04
Future|2050–SSP585	0.916 ± 0.004	0.66 ± 0.03
Future|2100–SSP126	0.914 ± 0.004	0.70 ± 0.03
Future|2100–SSP585	0.912 ± 0.004	0.70 ± 0.04

B		
Environmental variables	Percentages of contribution (%)	
Precipitations (mm)	45.9	
Vapor pressure (kPa)	28.4	
Temperature (°C)	20.6	
Solar radiations (kJ m^−2^ day^−1^)	2.4	
Isothermality	1.7	
Altitude (m)	0.6	
Diurnal	0.4	

The current predicted distribution of suitable habitats showed that *C. sowerbii* was present on all continents, excluding Antarctica, with hotspots in North America (USA and Canada), in South America (eastern part), and in Europe (Fig. 2B). However, suitable habitats were recorded in the native ranges of China, as well as in Japan and Australia. In contrast, areas in central Asia appear to be less suitable for *C. sowerbii* populations.

Precipitation, vapor pressure, and temperature were the most important variables influencing the suitable habitats for *C. sowerbii* (Table 1B). The response curves of the used variables had a wide range of temperatures (10 to 35 °C), with two optima at 22 and 35 °C (Supplementary Fig. S7). Regions with precipitations between ~ 200 and 2500 mm (optimum = 1000 mm) and a vapor pressure between 0.1 and 3.0 kPa (optimum = 0.5 kPa) were suitable for *C. sowerbii.* This hydrozoan species was observed over a large range of solar radiations (0 to 32,000 kJ m^−2^ day^−1^), with an optimum at 5000 kJ m^−2^ day^−1^.

**Figure 3:**
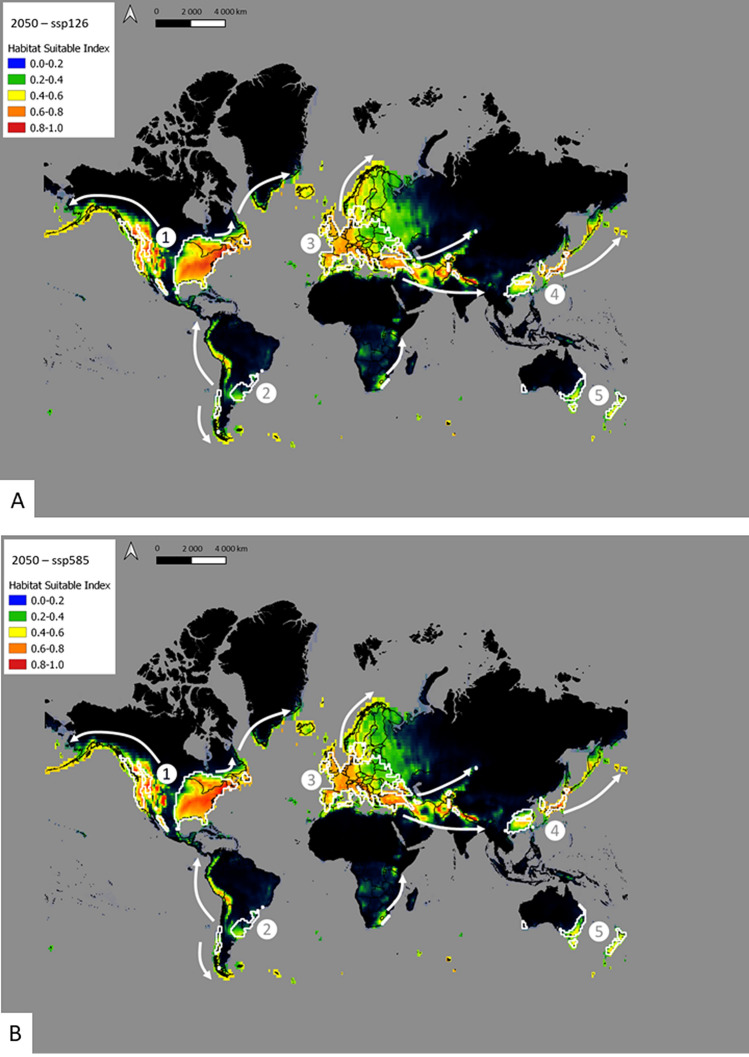
Distribution of *Craspedacusta sowerbii* under future climate conditions in 2050 in (**A**) lower limit (SSP126) scenario and (**B**) upper limit (SSP585) scenario. White arrows show the expansion of *C. sowerbii* from the main hotspots (indicated by numbers) observed in the current predictions (white areas). Map produced using QGIS version 3.16.3 software, https://www.qgis.org/fr/site/index.html.

**Figure 4:**
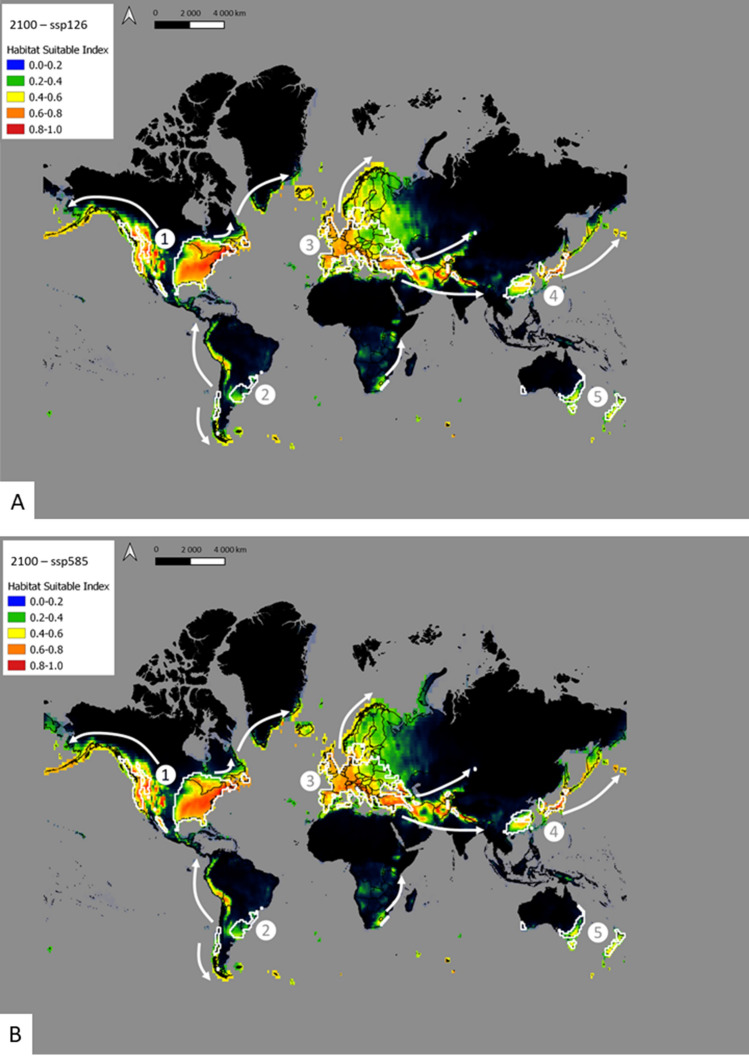
Distribution of *Craspedacusta sowerbii* under future climate conditions in 2100 in (**A**) lower limit (SSP126) scenario and (**B**) upper limit (SSP585) scenario. White arrows show the expansion of *C. sowerbii* from the main hotspots (indicated by numbers) observed in the current predictions (white areas). Map produced using QGIS version 3.16.3 software, https://www.qgis.org/fr/site/index.html.

Predictions of suitable habitats modeled for 2050 and 2100 as well as both scenarios (SSP126 and SSP585) showed highly similar patterns (Figs. 3 and 4). Maps based on the high limit scenario (SSP585) were similar and the main difference between 2050 and 2100 was the increase of Habitat Suitable Index (HSI) in 2100. The predicted distribution of *C. sowerbii* in 2050 and 2100 was considerably larger than at present (Figs. 3 and 4). In North America, a large expansion of *C. sowerbii* could be noticed, extending its current distribution 4000 km along coastal Alaska and the Aleutian Islands chain. From its hotspot in eastern South America, *C. sowerbii* was predicted to expand over a distance of 2000 km along the western coast to the north and 1000 km to the south. In Europe, *C. sowerbii* was already widely distributed, but its future distribution is predicted to extend also to northern colder regions (Norway, Sweden, Finland). A large expansion to the east (about 5000 km) was noticed from Europe to Asia (Iran, Pakistan, India). Another expansion of its distributional range is predicted in eastern Asia, where the species will spread over a distance of 4000 km from Japan to Russia (Kamchatka, Sakhalin, Kuril Islands chain). In Oceania, predictions for future scenarios showed no range expansion of the species, which remained widespread over the same areas defined in the current scenario.

**Figure 5:**
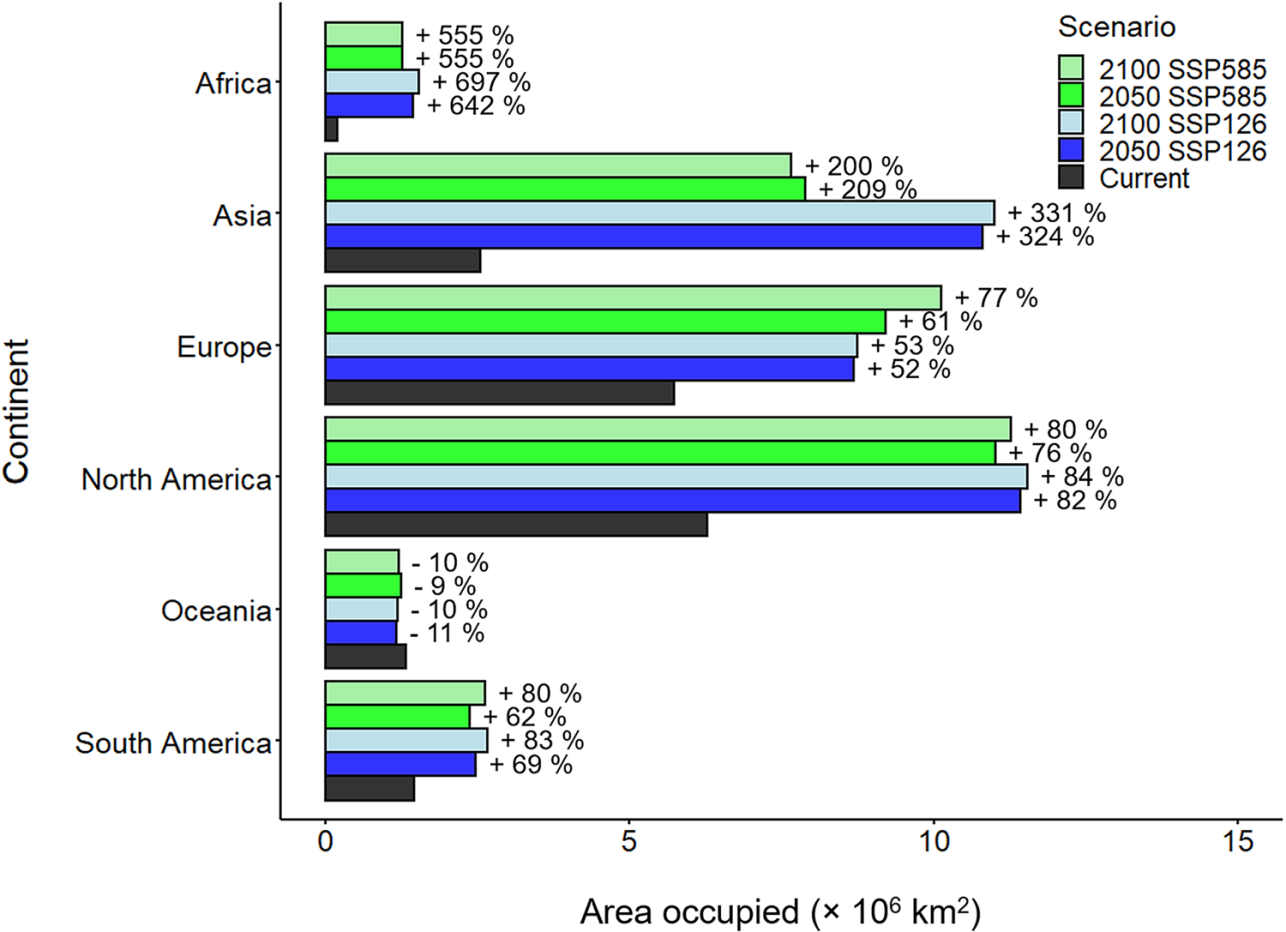
Estimated occupied areas (km^2^) by *Craspedacusta sowerbii* according to the current and future (2050 and 2100) scenarios.

The estimated occupied area by *C. sowerbii* for the current epochs showed highest values in North America (6,274,075 km^2^) and Europe (5,728,984 km^2^; Fig. 5), followed by Asia (2,550,718 km^2^), South America (1,458,056 km^2^), Oceania (1,315,739 km^2^), and Africa (192,173 km^2^). An expansion of the distributional range of *C. sowerbii* under future epochs (2050 and 2100) was observed for all continents, except Oceania. In 2050, the area covered by *C. sowerbii* was predicted to increase on average by 598 ± 62% in Africa (SSP126: 642%; SSP585: 555%) and by 266 ± 82% (SSP126: 324%; SSP585: 209%) in Asia. In North America, the forecasted expansion will be 79 ± 5% (SSP126: 82%; SSP585: 76%) and in Europe 56 ± 6% (SSP126: 52%; SSP585: 61%). In South America, the expansion of *C. sowerbii* was estimated in 2050 at 65 ± 4% (SSP126: 69%; SSP585: 62%). Interestingly, the area potentially inhabited by *C. sowerbii* in Oceania would decreased by 8 ± 4%. In 2100, the same pattern was observed for the lower limit (SSP126). While, the upper limit (SSP585) in 2050 and 2100 induced an expansion of *C. sowerbii,* but in lower proportions than the lower limit (SSP126).

## Discussion

The proliferation events of *Craspedacusta sowerbii* are irregular, unpredictable, and short-lived^19^, and mentions of this cryptic species are mainly based on citizens' observations. Thus, it is difficult to monitor this species locally and Species Distribution Models (SDMs), e.g., Maxent, are good tools to produce potential geographical distribution maps of freshwater species^20^. Identifying suitable habitats for highly invasive species such as *C. sowerbii* using SDMs is among one of the most critical targets and a current aim of conservation science^21^ providing powerful means in predicting distributional trajectories. Suitable habitats were identified beyond the current occurrence records strongly suggesting the limitation of current observations and expansion potential of this species. These results are consistent with the ability of non-indigenous species (NIS) to occupy and colonize new environments similar to those in their original ranges. The employed approach showed exceptional performance based on the omission rate and was confirmed by the test records for each scenario. The consistency of the model was also corroborated by high Area Under the Curve (AUC) values (> 0.9)^22^ and True Skilled Statistics (TSS) values^23^. Although there is always some degree of uncertainty inherently affecting modeling approaches^21,24,25^, SDMs represent one of the best methods to date in predicting the biological invasions and designing the management measures^26^. Among SDMs, Maxent is the most common model currently used to predict suitable habitats^27–29^ offering particular advantages for predicting rare species distributions. The good agreement between the *C. sowerbii* contemporary aquatic distribution and predictions provides for an excellent validating platform of models originally developed for terrestrial ecosystems^30^. When coupled with functional trait-based or fundamental niche-based approaches, findings of this study significantly improve our predictive abilities for current and future spatial aquatic NISs distributions^31,32^.

*Craspedacusta sowerbii* is currently and will in the future inhabit subtropical regions and the warm-temperate-cold belt in both hemispheres. Records of freshwater jellyfish are added every year^4^. Using this modeling approach, it was possible to present a current and future global distribution of *C. sowerbii*. In both future epochs (2050 and 2100), *C. sowerbii* showed a large expansion potential from a currently wide distribution to colder areas in northern and southern parts of the world. Except in Oceania where the range decreased, the expansion of *C. sowerbii* was observed for both future epochs even if the expansion was more pronounced in 2050 than in 2100. The decrease of suitable habitats can potentially be explained by a temperature exceeding the tolerance threshold of the species^33^. The data used in this study originate from the literature and are, in part, decades old, which implies that the environmental parameters of the specific observation site have potentially already changed. It is therefore essential to conduct new species monitoring programs on a national or international scales in order to overcome temporal differences. In this study, modeling was performed exclusively on medusa stage, which is easier to observe by researchers and the general public than the polyp stage, which is very small and cryptic. Our study recorded only observations of this species in medusa phase in pelagic zones. This new inventory can be supplemented by new observations of this species, offering a basis to determine whether the population will increase with currently ongoing climate change. It is also important to note that this new map only shows areas where medusa have been observed. The spatial distribution of *C. sowerbii* could be and likely is much more extensive if we would consider the polyp stage. Future increases in temperature may cause a greater recurrence of blooms of this species. The polyp stage can also be present without ever producing jellyfish, dispersing from the various resistance structures, and going unnoticed by researchers and the general public. Most likely, the distribution of polyps is much wider than that of jellyfish (currently and in the future). But in this study, the presence of the polyp stage can be considered as a proxy for the presence of the jellyfish stages.

This study shows that populations of *C. sowerbii* are mainly established in closed/current-reduced environments (e.g., lakes, ponds, basins, and water-filed quarries), which seem to be an asset for cnidarians as observed in the Palau archipelago where explosive population developments of the marine scyphozoan *Mastigias papua* and the cosmopolitan *Aurelia aurita* have been recorded^34,35^. *Craspedacusta sowerbii* is mainly observed in oligotrophic and shallow environments^36–39^ as well as in small (< 25 ha = 0.25 km^2^) eutrophic lakes^9,40^, isolated from rivers, restricting the supply of cold, rapidly-moving water that could limit the medusa budding. The shallow depth and high light intensity allow the water to reach temperatures > 25 °C, promoting asexual (and sexual) reproduction^11,41,42^. High temperature is necessary, but certainly not the only requirement^11,42^. The thermal ranges (4–35 °C) were consistent with the environmental ranges reported in the literature^43^. The optima at 22 and 35 °C obtained in this study were realists with data available in the literature, where an optimum at 19–20 °C for polyp growth and an optimum of 29–30 °C for medusa production were observed^11^. The solar radiation only contributed to a minor extent to the observed distribution pattern, consequently photodamage is not a limiting cause despite most often shallow water bodies and exposure to high radiation levels. *Craspedacusta sowerbii* can be exposed to sublethal and lethal amounts of ultraviolet radiation in very clear lakes, however, increased levels of dissolved organic carbon can act as protection, allowing *C. sowerbii* to survive even under high solar radiation levels^44^. Similarly, the deep-living scyphomedusa *Periphylla periphylla* undertakes high-amplitude diel vertical migrations (DVMs) in Norwegian fjords to prevent high light exposure^45^, which could soon alter because of climate change-facilitated increased turbidity. On the contrary, the freshwater jellyfish *Limnocnida tanganjicae* undertakes low-amplitude DVMs to minimize UV radiation exposure and thus photodamage^46^.

Although identifying the causes of the current expansion of *C. sowerbii* is beyond the scope of this study, it supports the idea that climate change, expressed as increased temperature and reduced/changed precipitation, is the most important environmental factor able to influence future suitable habitats for this species. It is also highly likely that anthropogenic activities (e.g., pollution, introductions, lake drainage, dams, etc.) may be factors that drive the distribution expansion of *C. sowerbii*. The same is true for biotic interactions as expressed by competition for food and space, predation, parasitism, commensalism, etc., which cannot be taken into consideration in the current model. However, in the context of global warming, strong summer heat waves will facilitate the recurrence of *C. sowerbii* proliferation. At 29 °C, a favorable temperature for medusa stage, the predation pressure of *C. sowerbii* on zooplankton can reach between 3.9% (umbrella diameter: < 1 mm) and 60% (umbrella diameter: 7–8 mm)^42^. During warm periods, medusae mainly consume small prey (0.2–2 mm) and kill larger (> 8 mm) organisms inducing a pressure on the zooplankton^9,47–49^ and likely affect the food web structure via trophic cascades^48^.

Passive population losses through advection in rivers, canals, and lakes connected to a river/canal are common to all freshwater plankton organisms. The solution to this problem is the production of a drought-resistant life cycle stage (podocyst) suitable for passive dispersion^13^. In the case of biological invasion, it is difficult to detect the exact vectors and pathways of introduction and dispersion^50^. Transport with aquatic plants, birds, fish, and humans is therefore one of the main suggested vectors for the introduction of *C. sowerbii*. *Craspedacusta sowerbii* began spreading throughout Japan soon after American troops entered the country after the Second World War^51^. Similarly, the species first appeared in Australia around 1950, but then spread rapidly^52^. Due to the intense commercial contacts with China, the invasion of Europe and North America probably began as early as the middle of the nineteenth century^13^. Tropical South America and Oceania were not colonized very much. The numerous occurrences of *C. sowerbii* in aquaria suggest that the “escape” from aquaria may mark the beginning of the conquest of a new area. Earliest South American data date back to the 1930s, with an aquarium observation^53^, quickly followed by observations in Chile, Brazil, Argentina, and Uruguay^13^. *Craspedacusta sowerbii* was found in an aquarium in Japan about six months after importing some aquatic plants from San Francisco^54^. It should be added that the only record of *C. sowerbii* in India before 1994^55^ was from an aquarium. In Quebec (Canada), the presence of freshwater jellyfish was suggested to be linked to the activity of boaters, who would seem to move the species from one water body to another^56^. Introduction to New Zealand probably came late, in line with the isolated position of the island group, and may not have occurred until the 1960s^57^. Southern Africa has also been colonized just recently: the first record was from the temperate Lake Midmar^58^, but records rapidly accumulated, and at least six sites were currently known in Morocco and South Africa.

Dumont^13^ first hypothesized about the role of bird’s migration in the aerial dispersion of *C. sowerbii* because of its drought-resistant resting polyps (podocysts). The current distribution of *C. sowerbii* indeed matches with the main world bird’s migration patterns^59^ with the proportion of migratory species increases with latitude (from equator to 80°N)^59,60^. Migrations of birds connect all continents, with eight main migratory routes^61^. In the Americas, there are three major migration routes connecting the north and south. Africa, Europe, and Asia are also inter-linked via three major migratory routes and these continents further connected to America via Eastern Asia, Europe, South Africa, and North America migratory routes. Oceania and Asia are connected via a migration route.

The migratory route hypothesis is corroborated by the observational data of *C. sowerbii*. It appears that the contemporary distribution and projected expansion could be further augmented by changes of migratory paths and their intensity, particularly in the northern hemisphere^59,61^. Phylogenetic studies of *C. sowerbii* populations have shown that the local populations were sourced from Chinese populations^62^ further illustrating the hypothesis of a connection via birds from Asia to North America, and from North America to Chile. As recently highlighted by Lüskow et al.^4^, there are two main widespread lineages of *C. sowerbii* distributed globally, which are present in China (together with other lineages) and in several countries in the Old and New World. Molecular information coverage is not yet sufficiently available to disentangle this species-complex, but in the near future we may learn that specimens so far considered as *C. sowerbii* belong to one or more species. The patchy and non-intuitive distribution of different lineages of *C. sowerbii* on a global scale^4^ may highlight the importance of migratory bird pathways. However, this hypothesis warrants further examination.

To conclude, the present study shows that global climate change will favor the expansion of *C. sowerbii* worldwide mainly facilitated by precipitation, vapor pressure, and temperature. Globally, this species will continue expanding to high-latitude regions in both hemispheres with diminishing rates of expansion by the end of the century.

## Methods

### Data collection

To study the global distribution of the occurrences of *Craspedacusta sowerbii* (polyps and medusa stages)*,* an extensive literature review was conducted to cover all known native and invaded areas. The search was performed in June 2021 using relevant keywords forming a simple search string ('*Craspedacusta sowerbii*' or '*Craspedacusta*' or 'Freshwater jellyfish') on the main peer-reviewed literature databases (Web of Science, Scopus, and Google Scholar).

The occurrences data in the United States of America were extracted from the Non-Indigenous Aquatic Species Database (U.S. Geological Survey, 2020, https://nas.er.usgs.gov/). Occurrence information were compiled using the database Global Biodiversity Information Facility (GBIF, https://www.gbif.org/). Information extracted from the literature and GBIF were organized and synthesized according to specific criteria, such as geographic area (continent and country), GPS coordinates, habitat types, with a complete list of studies (Table S1) and in free access in PANGAEA^64^.

### Habitat types

To trace the evolution of number of records of *C. sowerbii,* the date of first record was extracted and the cumulative number of records as a function of time was visualized (R version 3.6.0). With the aim to investigate the affinity of *C. sowerbii* with specific habitat types, all information about the habitat types (i.e., closed/open, artificial/natural, rivers, canals, ponds, lakes, etc.) available in the scientific literature were compiled. The number of records as a function of habitat types was plotted. To comprehend the altitude ranges of *C. sowerbii*, altitude data were extracted from the elevation layer downloaded from WorldClim (version 2.1, spatial resolution 85 km^2^; http://www.worldclim.org/; accessed in June 2021)^65^ using QGIS software (version 3.16.3; https://www.qgis.org/fr/site/index.html).

### Environmental data

High-resolution inland water data are currently not available on a global scale. However, the climate variables, in conjunction with physical habitat data, could serve as reasonable surrogates for inland water variables^66^. These distal variables were often causally related to proximal factors in freshwater species distributions (e.g., air temperature directly affects water temperature, altitude and slope directly influence channel morphology)^65,67^. Bioclimatic variables used to perform Maximum entropy (Maxent) analyses were extracted from WorldClim at spatial resolution of 2.5 arc minutes. The variables include the means and ranges of temperature, precipitation, water vapor pressure, and solar radiation, and characterized the dimensions of climate considered particularly relevant in determining species distributions (Table S2). Prior to model fitting, autocorrelation between environmental variables was checked to prevent inclusion of other correlated variables by applying the function 'rcorr' in the package 'corrplot’^68^ in R version 3.6.0^69^. Generally, temperature was related with precipitation and solar radiation, and vapor pressure with solar radiation and precipitation (see for details Supplementary Fig. S1). Altitude data were included in the model as well.

To assess the distribution patterns of *C. sowerbii* in response to future climate change, models were run for the years 2050 and 2100. Data used for the two future epochs (2050 and 2100) were taken from climate models BCCCSM2-MR, the Beijing Climate Center. The lower and the upper limits of the Shared Socioeconomic Pathways (SSPs; SSP126 and SSP585) were used for better prediction of future conditions. Currently, SSPs are used by the Intergovernmental Panel on Climate Change (IPCC) in the 6th Assessment Report. These data were obtained from the Coupled Model Intercomparison Project Phase 6 (CMIP6)^70^.

### Habitat suitability modeling

Maximum entropy (Maxent; http://www.cs.princeton.edu/*schapire/maxent/, version 3.4.4.)^71^ models are one of the most widely used Species Distribution Model (SDM) algorithms, relating species presence to corresponding environmental variables and allowing to estimate suitable habitats for the target species^71,72^. Maxent has proven to be more effective than other methods due to a higher predictive accuracy, making it more reliable in modeling range shifts under future climate change scenarios^73^. It has been particularly useful in predicting the geographic distribution of Non-indigenous species (NIS) in the context of climate change^74–76^. The cleaned occurrence data (duplicates in the same area have been removed) were used to predict habitat suitability^71^*.* Default parameters, including logistic output, regularization multiplier 1 and 10,000 background points were used. The continuous fit predictions were converted to binary predictions using the command 'threshold equal training sensitivity and specificity' in Maxent. “Sensitivity” is the probability that a model correctly predicts an observation (true positive rate), whereas 'specificity' is the probability that a known absence is correctly predicted (true negative rate). This was the most reliable threshold to minimize the absolute difference between sensitivity and specificity^77^, balancing the accuracy of areas modeled correctly as present/absent in the training and test data. Specifically, at this threshold, the probability of missing an appropriate distribution and assigning an inappropriate distribution is the same.

Model evaluation was performed via the random test percentage, dividing the dataset into training (70%) and test (30%) data, subsamples (equal to the number of observations), and 5000 iterations, using the easily interpretable logistic output format with habitat fit values. Maxent models generate an estimate of the probability of species occurrence ranging from 0 (i.e., unsuitable habitat) to 1 (i.e., optimal habitat) representing the distribution in geographic space of the suitable habitat (i.e., Habitat Suitability Index (HSI))^72,78^. Maps were produced and the areas (km^2^) covered by *C. sowerbii* for the HSI > 0.4 were calculated using QGIS version 3.16.3 software.

### Model evaluation

The performance model was evaluated by calculating the Area Under the Curve (AUC) of the Receiving Operator Characteristic (ROC), a measure for the discrimination capacity of the generated models^79^. Models with an AUC of 0.5 correlated with the expected performance of a random classifier, [0.7–0.8] were considered acceptable predictions, [0.8–0.9] were excellent predictions, and > 0.9 were exceptional predictions^22^. The True Skilled Statistics (TSS) was also calculated. TSS values ~ 0 (or < 0) represent distributions no better than random, while values equal to 1 represent a perfect agreement between the observed and the predicted distribution^23^. From these scenarios, the HSI was calculated for each time step and presented as geographic forecast maps. Jackknife tests were produced by the model to measure the marginal response curves of environmental variables to predict the probability of occurrence.

## Supplementary Information

Supplementary Information.
