# Supplementary material for: Predicting the current and future global distribution of the invasive freshwater hydrozoan Craspedacusta sowerbii
Source: Sci Rep. 2021 Nov 29;11:23099. doi: 10.1038/s41598-021-02525-3 (PMC8629981; doi:10.1038/s41598-021-02525-3)
Supplement: Supplementary file 1 — Supplementary Information. [file 41598_2021_2525_MOESM1_ESM.docx]

# Supplementary materials

Table S1. Information extracted from the literature to summarize in the dataset.

| **Type of information** | **Details** |
| --- | --- |
| ID | ID of each record |
| Year | First year of recording |
| Continent | Continent’s name |
| Region / State | Extracted from literature |
| Nearest city | If mentioned in the literature |
| Site name | If mentioned in the literature |
| Latitude | In decimal |
| Longitude | In decimal |
| Height above sea level | Altitude (m) extracted from WorldClim |
| Habitat type 1 | Categorical variable (Basin, Canal, Lake, ND, Pond, Reservoir, River, Water-filled quarry) |
| Habitat type 2 | Categorical variable (Artificial, Natural, ND) |
| Habitat type 3 | Categorical variable (Closed, ND, Open) |
| Reference | Citation of literature |
| ND | Data not available |

Table S2. Types of variables extracted from WorldClim (time period = 1970-2000).

| **Variable code** | **Type of variable** |
| --- | --- |
| BIO1 | Annual Mean Temperature |
| BIO2 | Mean Diurnal Range (Mean of monthly (max temp - min temp)) |
| BIO3 | Isothermality (BIO2/BIO7) (×100) (day-to-night temperature oscillation) |
| BIO4 | Temperature Seasonality (standard deviation ×100) |
| BIO5 | Max Temperature of Warmest Month |
| BIO6 | Min Temperature of Coldest Month |
| BIO7 | Temperature Annual Range (BIO5-BIO6) |
| BIO8 | Mean Temperature of Wettest Quarter |
| BIO9 | Mean Temperature of Driest Quarter |
| BIO10 | Mean Temperature of Warmest Quarter |
| BIO11 | Mean Temperature of Coldest Quarter |
| BIO12 | Annual Precipitation |
| BIO13 | Precipitation of Wettest Month |
| BIO14 | Precipitation of Driest Month |
| BIO15 | Precipitation Seasonality (Coefficient of Variation) |
| BIO16 | Precipitation of Wettest Quarter |
| BIO17 | Precipitation of Driest Quarter |
| BIO18 | Precipitation of Warmest Quarter |
| BIO19 | Precipitation of Coldest Quarter |
| Altitude | Altitude (m) |
| SRAD | Solar radiation (kJ m^-2^ day^-1^) |
| VAPR | Water vapor pressure (kPa) |


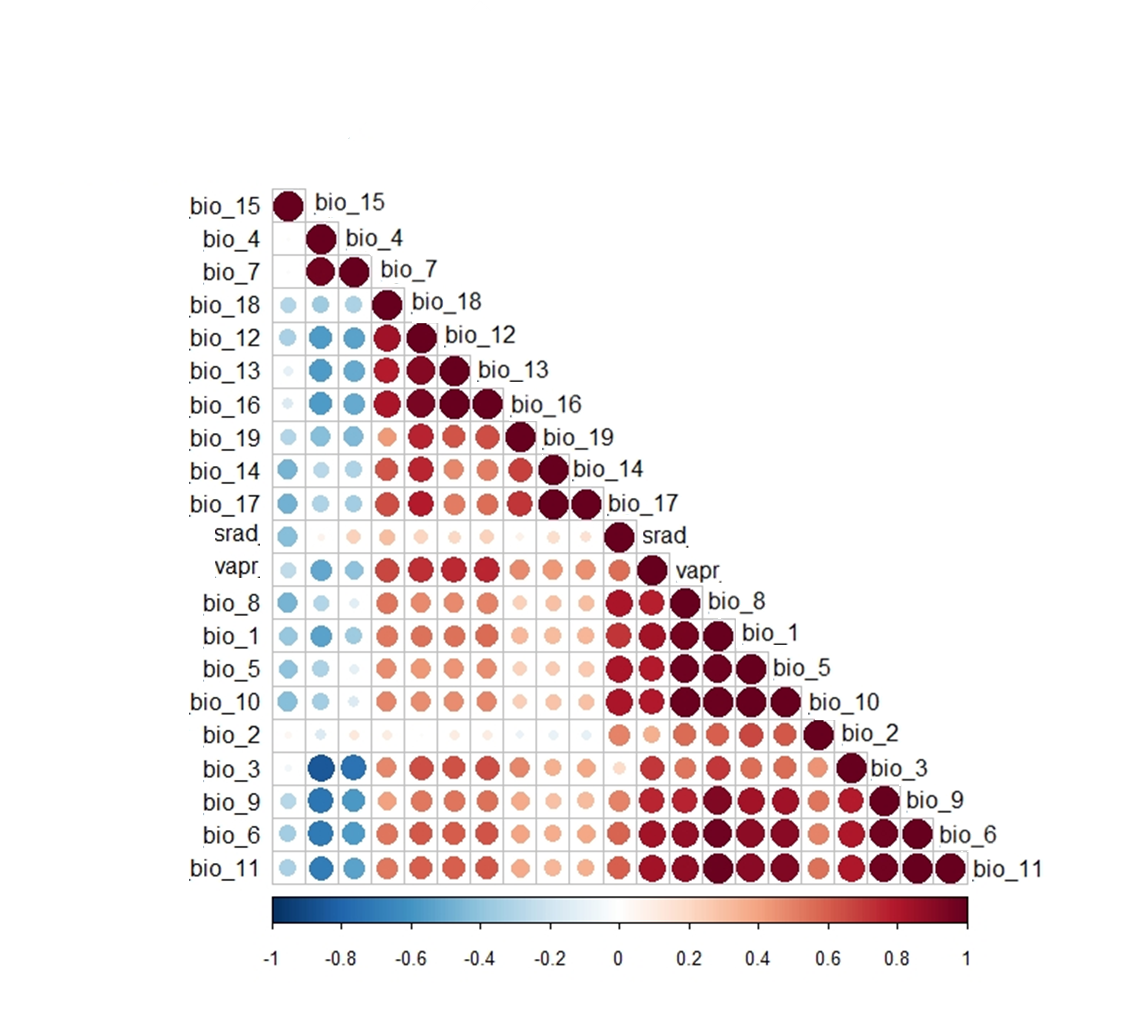


Figure S1. Scatterplot matrix of the environmental variables considered in current scenario represented to detect correlations between variables. The color scale is represented absolute Pearson correlations.


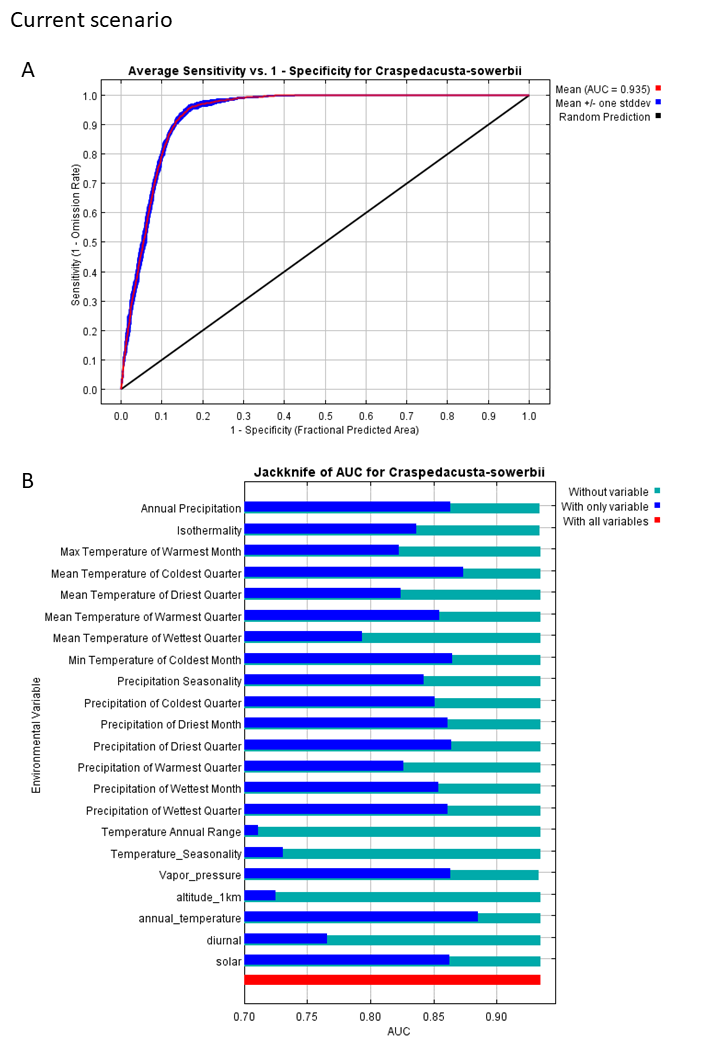


Figure S2. A) ROC (Receiver Operating Characteristic) curves for *Craspedacusta sowerbii* current models; AUC: Area Under the Curve. B) Jackknife tests of variable importance for *C. sowerbii* current models.


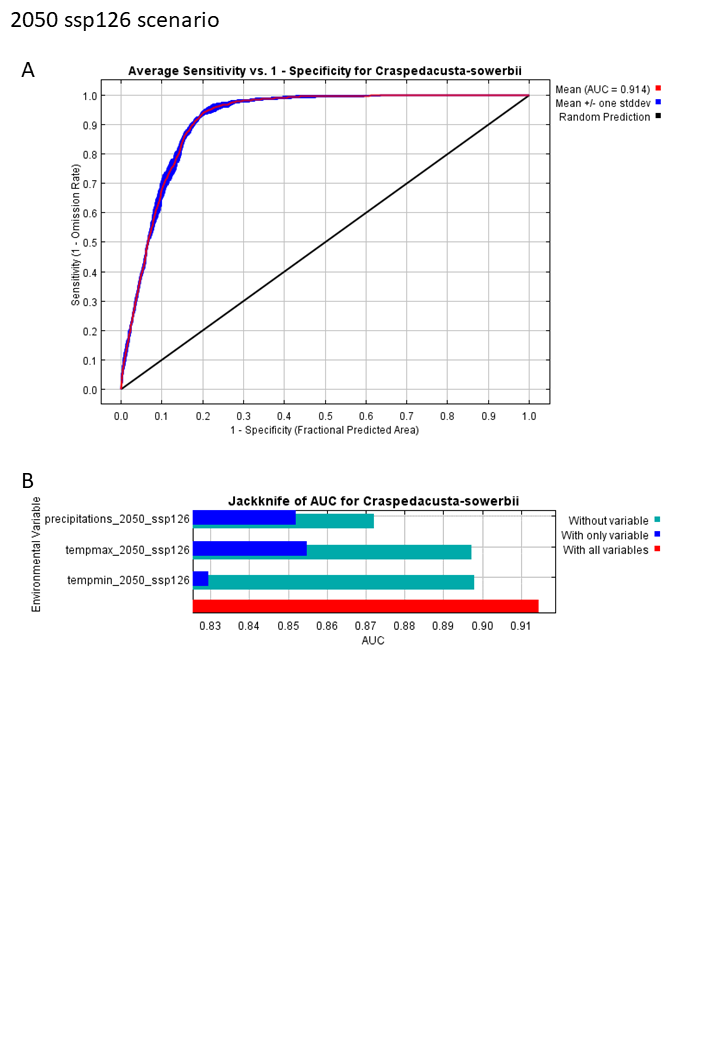


Figure S3 A) ROC (Receiver Operating Characteristic) curves for *Craspedacusta sowerbii* 2050 SSP126 models; AUC: Area Under the Curve. B) Jackknife tests of variable importance for *C. sowerbii* 2050 SSP126 models.


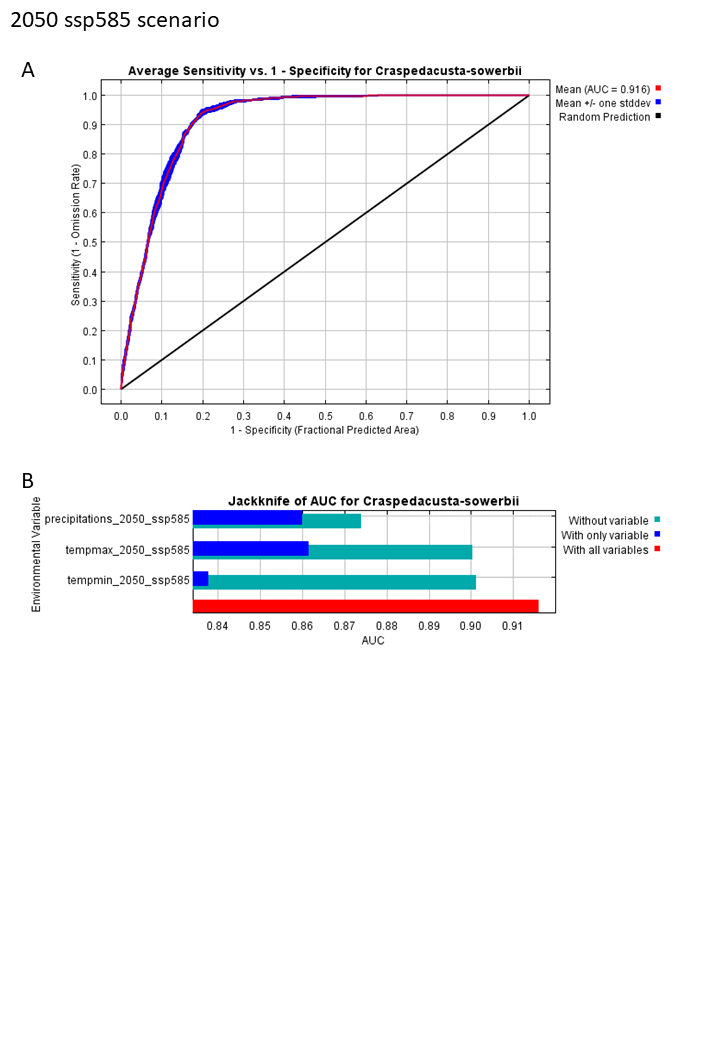


Figure S4. A) ROC (Receiver Operating Characteristic) curves for *Craspedacusta sowerbii* 2050 SSP585 models; AUC: Area Under the Curve. B) Jackknife tests of variable importance for *C. sowerbii* 2050 SSP585 models.


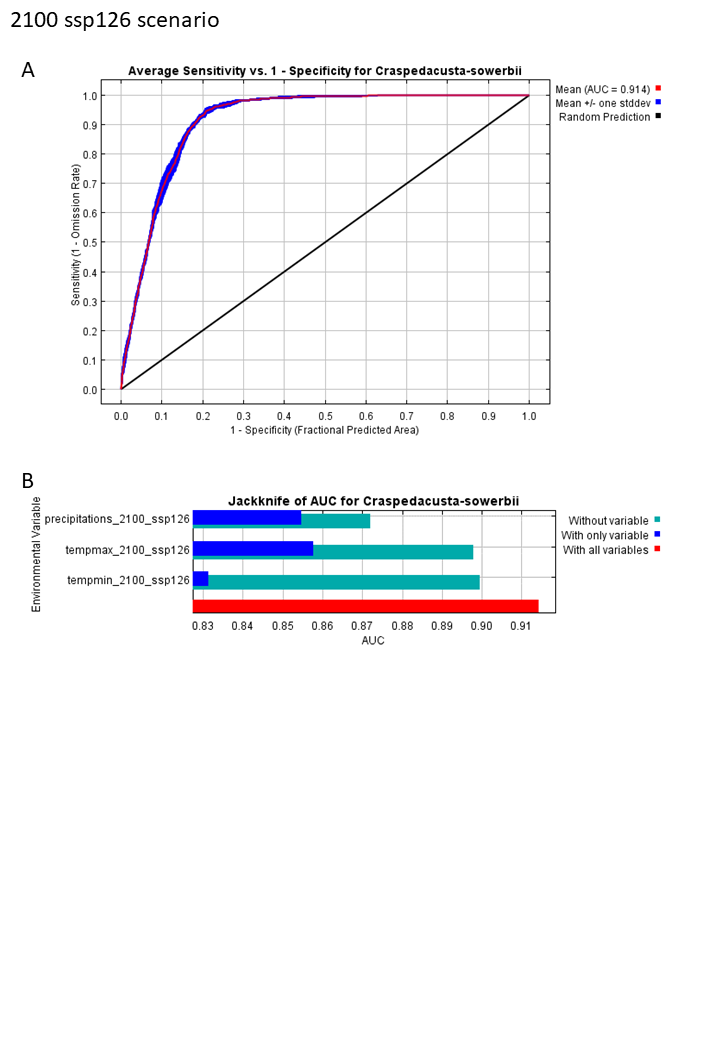


Figure S5. A) ROC (Receiver Operating Characteristic) curves for *Craspedacusta sowerbii* 2100 SSP126 models; AUC: Area Under the Curve. B) Jackknife tests of variable importance for *C. sowerbii* 2100 SSP126 models.


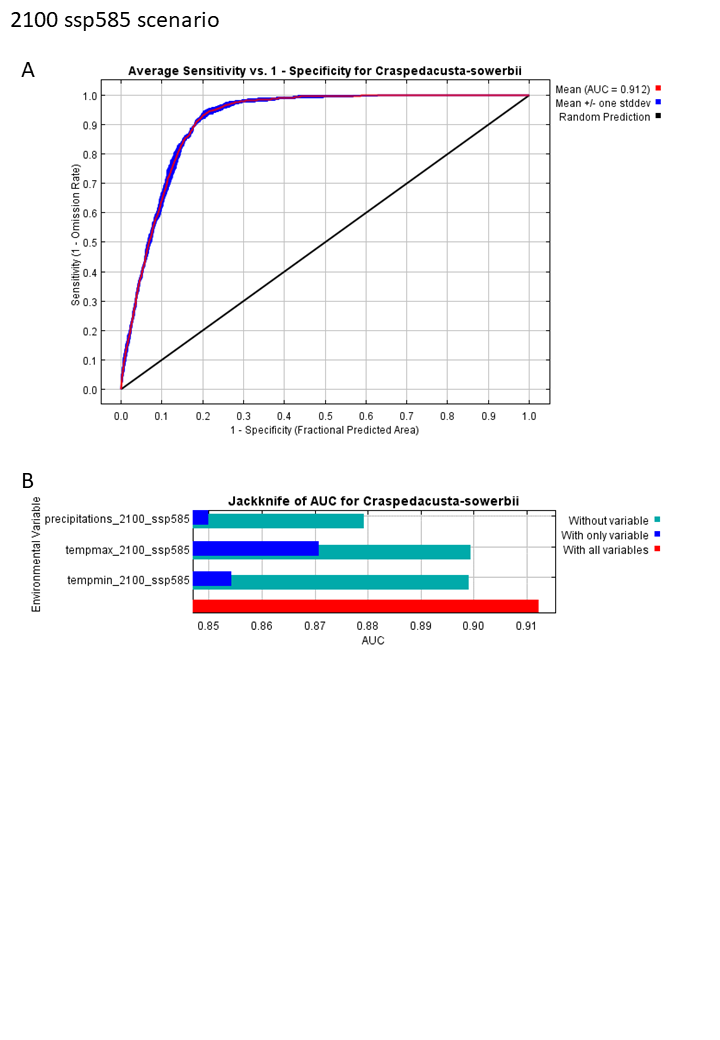


Figure S6. A) ROC (Receiver Operating Characteristic) curves for *Craspedacusta sowerbii* 2100 SSP585 models; AUC: Area Under the Curve. B) Jackknife tests of variable importance for *C. sowerbii* 2100 SSP585 models.


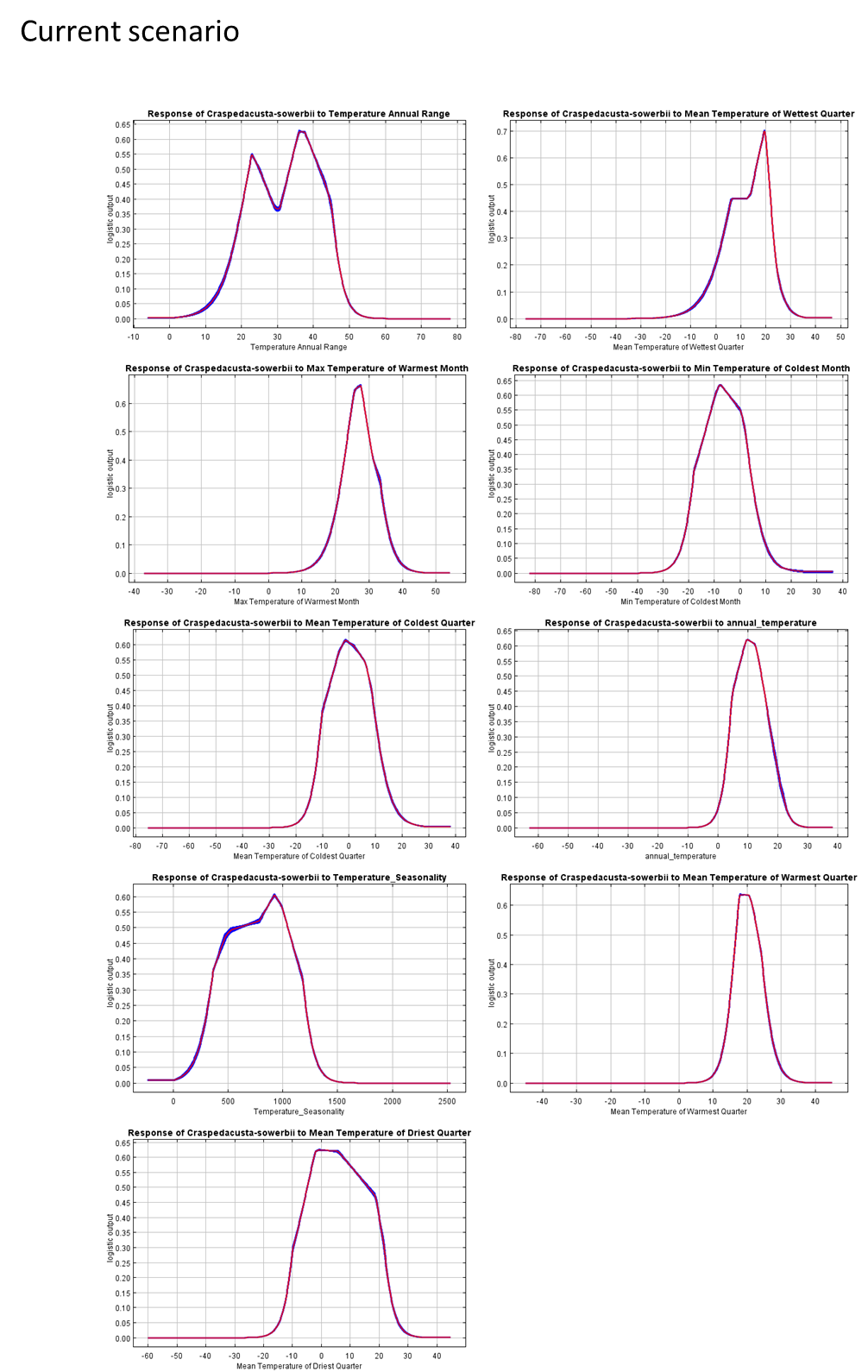


Figure S7. Partial dependence curves of the marginal response for *Craspedacusta sowerbii* to each variable for the current model.


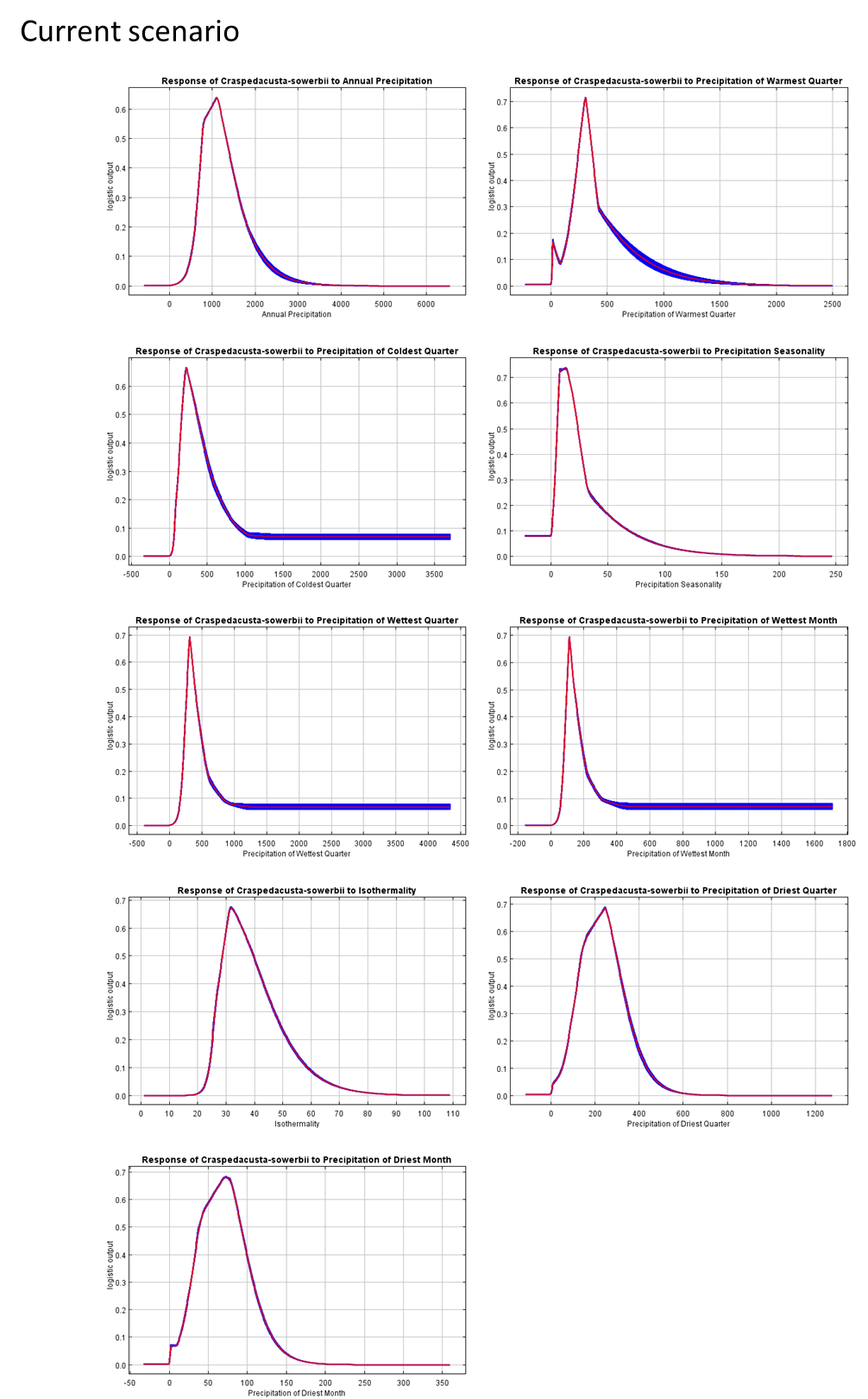


Figure S7. Continue


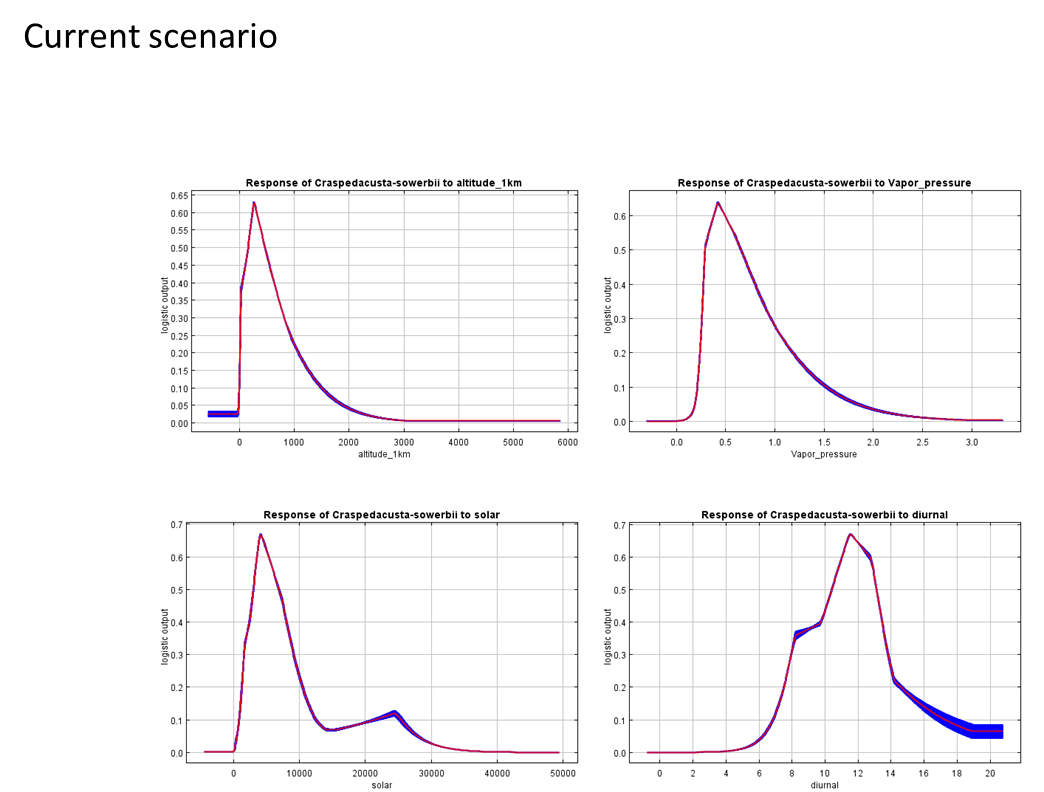
 Figure S7. Continue


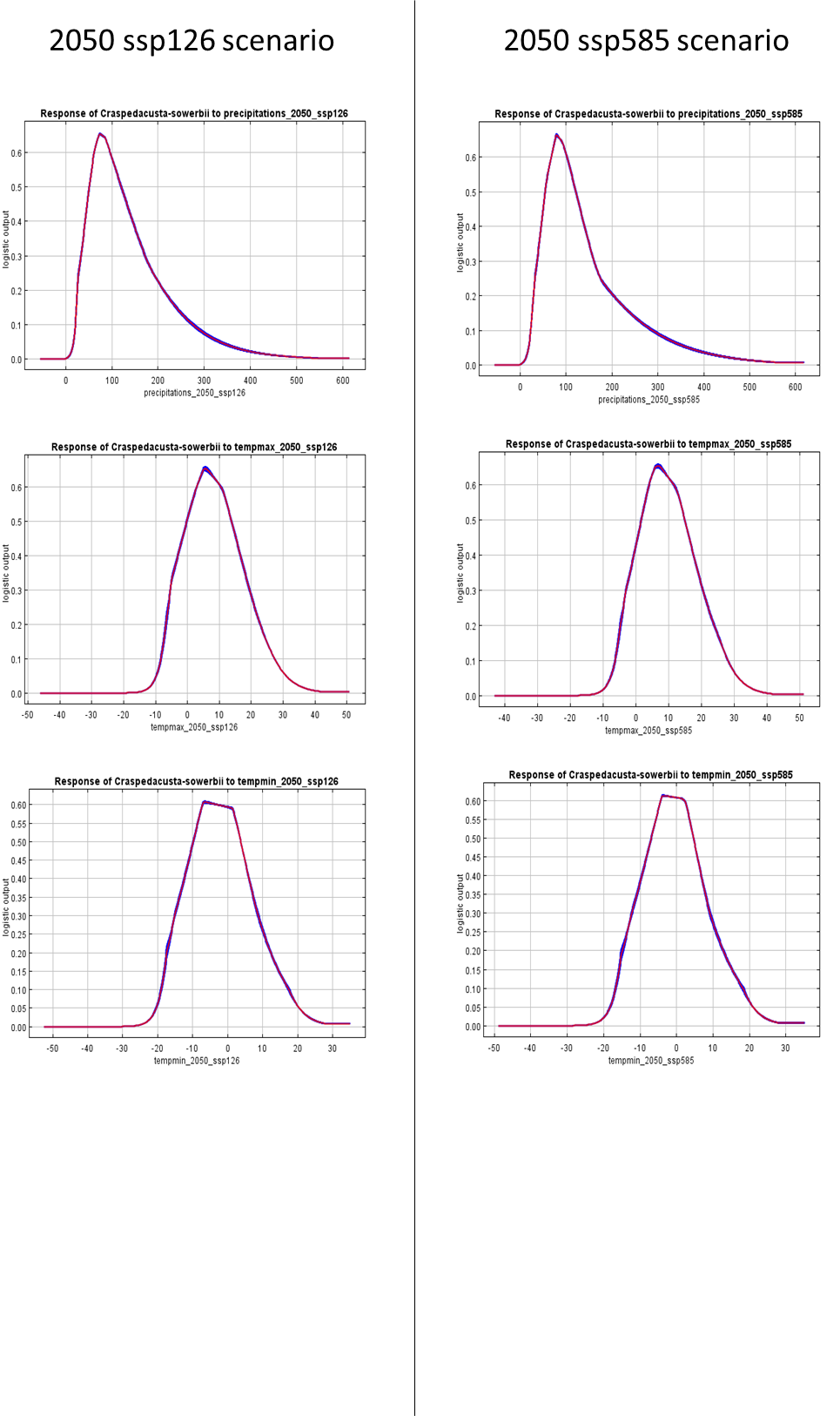


Figure S8. Partial dependence curves of the marginal response for *Craspedacusta sowerbii* to each variable for the 2050 (SSP126, SSP585) model.


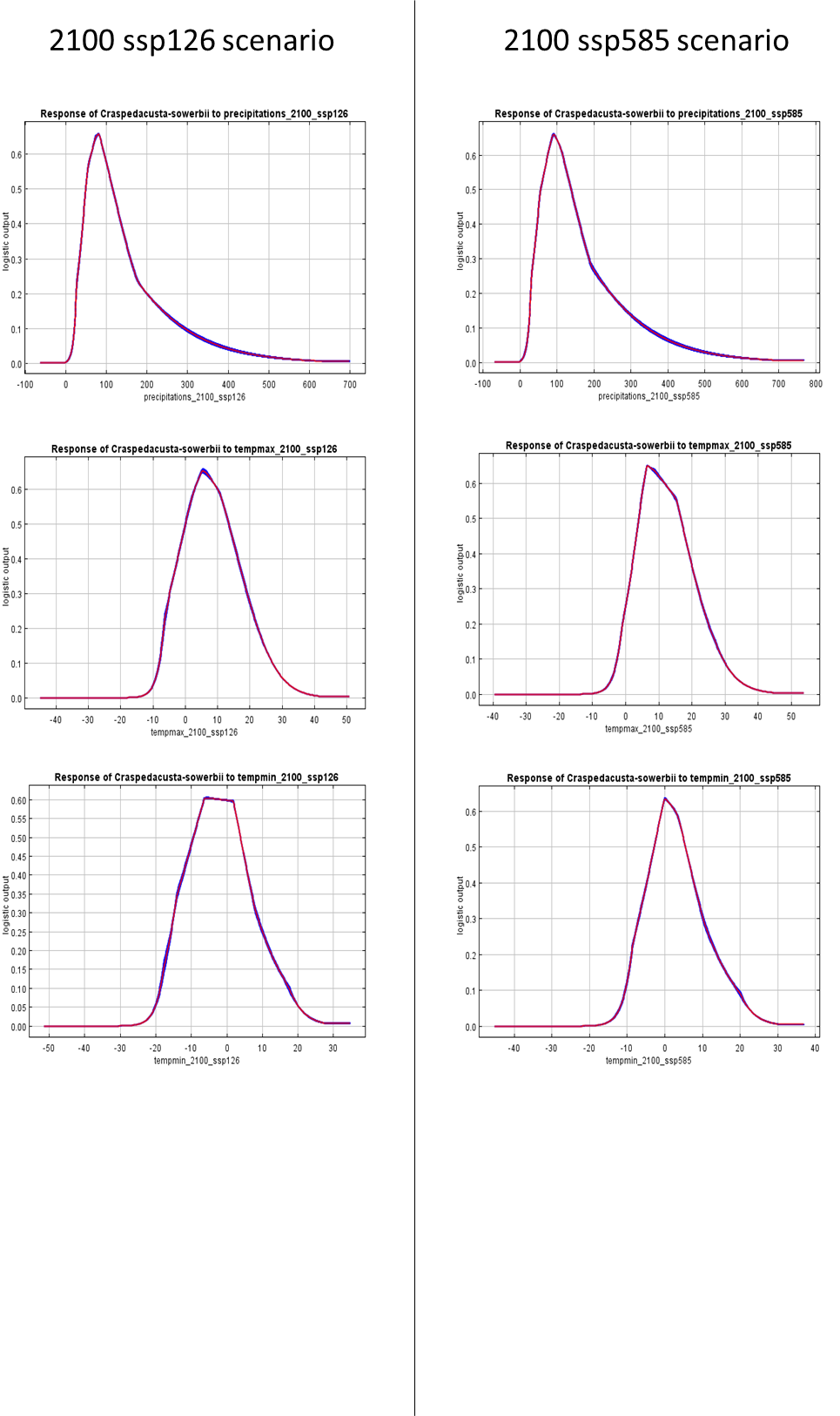


Figure S9. Partial dependence curves of the marginal response for *Craspedacusta sowerbii* to each variable for the 2100 (SSP126, SSP585) model.
